# Enhanced detection of surface deformations in LPBF using deep convolutional neural networks and transfer learning from a porosity model

**DOI:** 10.1038/s41598-024-76445-3

**Published:** 2024-11-06

**Authors:** Muhammad Ayub Ansari, Andrew Crampton, Samer Mohammed Jaber Mubarak

**Affiliations:** 1 School of Computing and Engineering, University of Huddersfield, Huddersfield, HD1 3DH UK; 2 University of Baghdad, Baghdad, 10071 Iraq

**Keywords:** Mechanical engineering, Computer science

## Abstract

Our previous research papers have shown the potential of deep-learning models for real-time detection and control of porosity defects in 3D printing, specifically in the laser powder bed fusion (LPBF) process. Extending these models to identify other defects like surface deformation poses a challenge due to the scarcity of available data. This study introduces the use of Transfer Learning (TL) to train models on limited data for high accuracy in detecting surface deformations, marking the first attempt to apply a model trained on one defect type to another. Our approach demonstrates the power of transfer learning in adapting a model known for porosity detection in LPBF to identify surface deformations with high accuracy (94%), matching the performance of the best existing models but with significantly less complexity. This results in faster training and evaluation, ideal for real-time systems with limited computing capabilities. We further employed Gradient-weighted Class Activation Mapping (Grad-CAM) to visualize the model’s decision-making, highlighting the areas influencing defect detection. This step is vital for developing a trustworthy model, showcasing the effectiveness of our approach in broadening the model’s applicability while ensuring reliability and efficiency.

## Introduction

Laser Powder Bed Fusion (LPBF) is a leading technique in additive metal printing, known for its advantages over traditional manufacturing methods. However, the lack of a real-time defect monitoring system often leads to costly post-production quality checks. Machine learning (ML), particularly Convolutional Neural Networks (CNNs), has shown promise in addressing this challenge by automating defect detection. Despite this potential, ML’s effectiveness is hindered by data scarcity, high data acquisition costs, and the complex nature of LPBF images, which contain minute defects difficult to label and identify^1,2^.

Convolutional Neural Networks (CNNs) are highly effective for image classification tasks due to their ability to extract features automatically and reduce feature dimensionality through pooling. In the context of LPBF, CNNs have been instrumental in identifying various material properties and detecting anomalies during the printing process. Different morphological characteristics, such as the sphere, needle, petal and polyhedron of Aluminium (Al) and Iron (Fe) alloys, were detected by employing a deep CNN^3^. Zheng et al.^4^ emphasised the importance of pre-processing the data for CNNs and constructed a multi-channel CNN to predict the material properties. Yuan et al.^5^ trained a CNN that predicted, in real-time, the laser track width, its standard deviation, and track continuity from the in-situ video. Ansari et al.^6^ detected porosity from powder bed images with an accuracy of 97% using CNNs. Mi et al.^7^ proposed a lightweight, deep CNN to detect different geometric properties of the melt pool and spatters from the in-situ images with an accuracy of 94.71%. Larsen and Hooper^8^ employed a deep, semi-supervised solution to distinguish between optimal process settings and defective settings and successfully detected 0.2% porosity with an Area Under the Curve (AUC) metric value of 0.999. Ansari et al.^9^ employed a CNN-based solution to identify surface deformation from powder bed images with 99% accuracy.

Using CNNs to detect micro-defects in LPBF powder bed images poses significant challenges. Training a CNN from the ground up demands a large dataset, which is difficult and costly to obtain in LPBF contexts. In image classification, the objects are assumed to be visible and cover a significant portion of the image^10–12^. Powder bed images, unlike MNIST and COCO images, feature micro-scale defects, leading to a small object-to-image ratio, requiring specialised data labelling skills. This situation mirrors the challenges found in classifying images of tiny objects, where extensive, expertly labelled datasets are necessary, underlining the complexities of accurately identifying LPBF defects^13^.

To navigate these challenges, this paper explores the use of transfer learning (TL), a method that applies knowledge gained from one task to another. TL has been very successful in numerous disciplines, especially medical research where breast cancer, brain tumour and Alzheimer’s disease have been diagnosed using TL on pre-trained models^14–17^. While TL has proven successful in various fields, its application in LPBF defect detection remains under-explored. Few studies reported in the literature use TL to solve LPBF problems. Li et al.^18^ successfully used the VGG-16 model to detect porosity in powder bed images with 99.89% accuracy, and demonstrated that applying TL could halve the training time with 99.27% accuracy. Pandiyan et al.^19^ successfully transferred the learning of VGG-16 and ResNet-18-based CNN solutions in identifying balling, lack-of-fusion, keyhole pores, and conduction mode defects from Stainless Steel (316L) to Bronze (CuSn8) metal powders using acoustic emissions signal data. Fischer et al.^20^ used a high-quality recoater-based line sensor (6µm/pixel) to capture defects like balling, spatter, scatter powder, incomplete spreading, protruding part, groove, and ridge, on images using various light settings. The CNN-based solution used a pre-trained Xception model using transfer learning and achieved the best accuracy of 99.15% and F1-score of 99.71% on the best lighting and image resolution settings. Although the accuracy of general purpose, pre-trained models used in the literature is excellent, they have millions of parameters with a poor prediction time. Fischer et al.^20^ suggested using a shallower CNN architecture as a future extension of their work. Similarly, Pandiyan et al.^19^ in their experiments also emphasised the importance of smaller-sized models as they are time-efficient and require less computational resources for deployment. Our work focuses on demonstrating the unsuitability of generic pre-trained models for LPBF defect detection due to their large size and slow prediction times. Instead, we advocate for a custom-built, efficient CNN model trained specifically for this purpose. We present a novel approach that leverages TL to adapt a model originally trained to detect porosity defects to identify surface deformations. This method not only offers a more secure and efficient solution by enabling on-device processing but also circumvents the data privacy and sharing concerns associated with cloud-based systems.

In this paper, we shall,

1. Highlight the limitations of general-purpose, pre-trained models for LPBF defect detection.
2. Demonstrate how to adapt domain-specific pre-trained CNN models to detect different LPBF defects through transfer learning.
3. Provide guidance to companies on developing their own tailored defect detection systems without relying on generic models.

We build upon our previous work on detecting porosity and surface deformation, showing how a model trained for porosity^6^ can be effectively optimized for surface deformation^9^ detection through transfer learning. Specifically, we construct a CNN and train it from scratch to classify porosity defects. We then transfer the learned behaviours (filters, weights, training parameters, etc.) and further optimise it to accurately model the surface deformation data set. To evaluate, compare and contrast the effectiveness of this approach, we also take state-of-the-art (SOTA), pre-trained models: VGG16, MobileNet, Xception, EfficientNetB1 and Inception ResNet V2 and fine-tune them on the same surface deformation data using the same transfer learning approach. Our findings reveal that this approach outperforms state-of-the-art models in terms of training parameters and prediction time, providing significant insights for the additive manufacturing community on creating efficient ML models for micro-defect detection. This paper not only presents a practical solution to the challenges of LPBF defect detection but also sets the groundwork for future research in domain-specific transfer learning.

The rest of the paper is organised as follows. Section two explains the experimental design. Section three presents the methodology, discussing the fine-tuning strategies and transfer learning approaches. Results and a critical evaluation of experiments are presented in section four. Section five concludes the paper with final remarks.

## Experimental design

Transfer learning^21^ utilises previously learnt knowledge to solve new (related) tasks. Among the three variations of the TL method, we employed inductive learning for our experiments because the source and target tasks are different. The source task is required to identify porosity in LPBF images, whereas the target task is to recognise surface deformation defects from the powder bed images.

### Data sets

The experiments were based on a porosity and surface deformation data set. The porosity data set constitutes the source domain, and the surface deformation data is referred to as the target domain.

#### Porosity data set

**Fig. 1:**
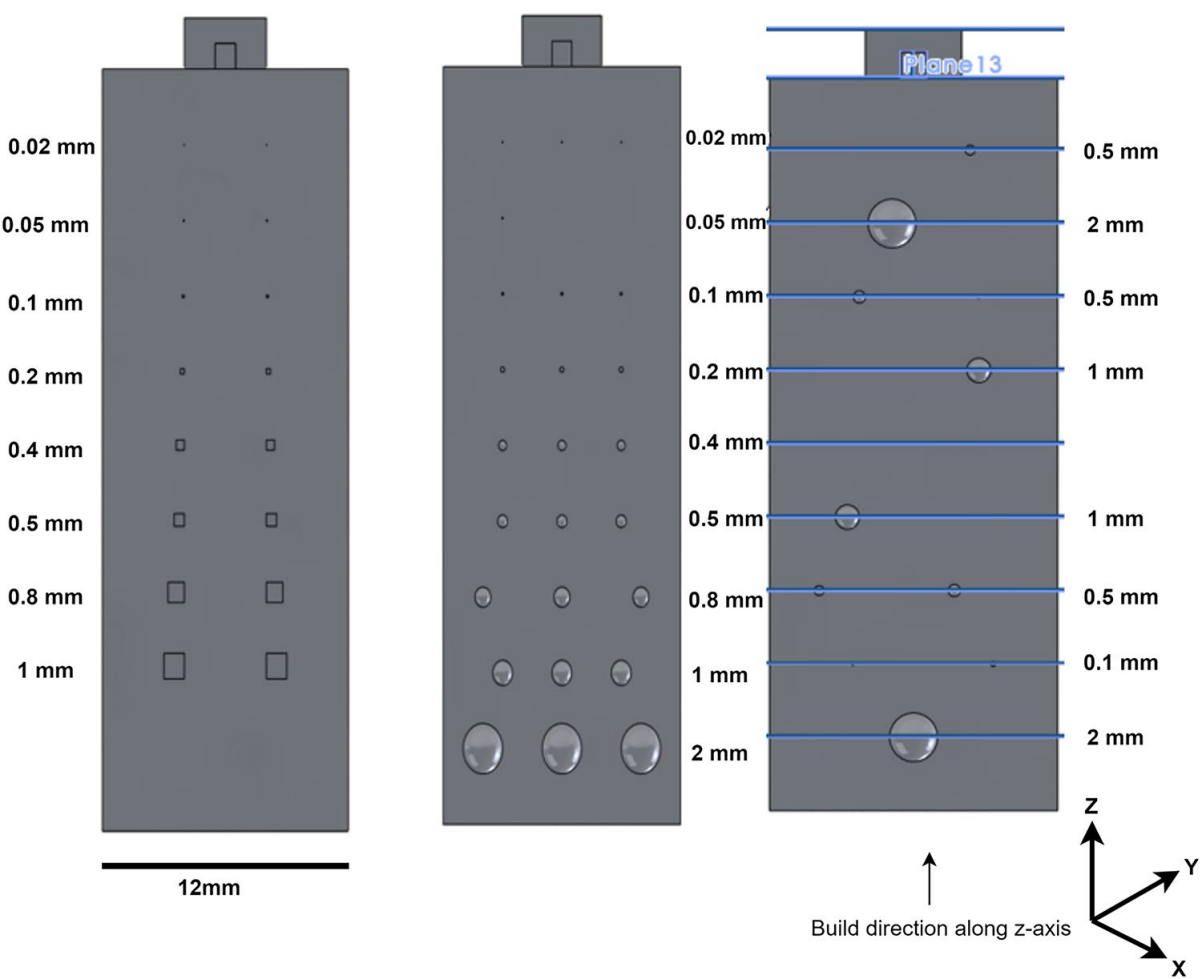
Computer aided design of three cylinders.

The porosity data set consists of three cylinders, each with a height of 30 mm and a diameter of 12 mm, as shown in Fig. 1. The cylinders were designed with seeded pores. The pores created in the test cylinders varied from 20 µm to 2 mm. B1 and B2 cylinders had circular pores, whereas B3 had cubical pores. The cylinders were produced using an SLM500HL machine (SLM Solutions, Germany) from Aluminium A20X powder with 20–53 µm size distribution. The temperature of the aluminium powder was maintained at $150\,^\circ$C throughout the build process. The process parameters used were 360 W laser power, 1500 mm/s scan speed, 100 µm scan spacing, and a layer thickness of 30 µm. The experiments were carried out using the stripe scan strategy.

In total, 2889 images were extracted from the printing job. However, the data was highly imbalanced. There were 2386 non-porosity images and only 311 porosity images. The imbalanced data set would result in biased model training. Therefore we employed data augmentation methods to over-sample the porosity images. Data augmentation is a well-established and effective way to combat class imbalance using affine transformations (zoom, shear, flips, mirror and colour perturbation, translation)^22^. The final, balanced porosity data set contained 5135 images, of which 2578 were non-porosity, and 2557 were porosity. For more details, the authors refer the interested reader to their previous work^6^.

#### Surface deformation data set

**Fig. 2:**
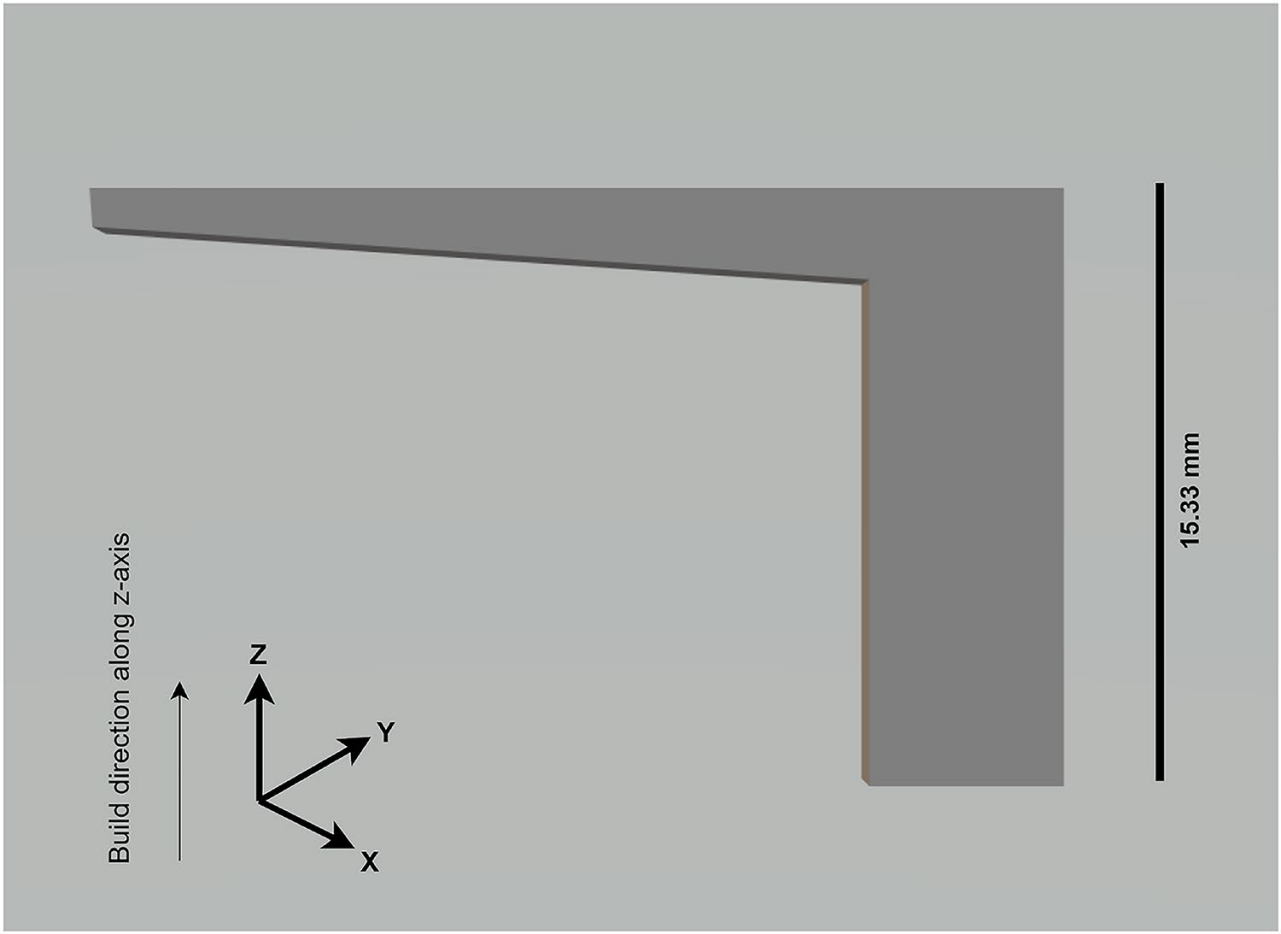
CAD design of the test specimen.

The experimental approach to capture data in the new domain carefully aimed to induce surface deformation using geometry variation. We designed thirteen bars with an overhang geometry for the surface deformation defects. The temperature gradient at the overhang region caused surface deformation defects during printing. The test specimens were printed using AlSi10Mg metal powder on an EOS M290 machine with default parameter settings. During the printing process, the other standard settings were: layer thickness of 30µm, laser power of 370 W, hatch distance of 0.19 mm, and a scan speed of 1300 m/s. The test specimens consisted of 511 layers and a height of 15.33 mm. The powder bed images were captured with cameras and sensors in an EOS printer that photographed each layer before and after laser exposure. The design of a bar is shown in Fig. 2.

A total of 5832 images corresponding to 486 layers were captured during the printing process. However, only a fraction of the layers (15%) contained surface deformation defects. The LPBF data is identical in many respects to the medical domain, where a significant class imbalance is notable. For balanced and unbiased learning, we kept only fourteen defect layers after the surface deformation and fourteen non-defect layers before the surface deformation defect. The final data set consisted of 336 images, of which 168 were normal and 168 had surface deformation defects. This data set is used as the target data set for transfer learning. The relatively small amount of training data highlights an essential need to deploy transfer learning and utilise pre-trained models as a starting point. The number of samples obtained would not be sufficient to practically train a deep CNN from scratch.

### CNN overview

A convolutional neural network (CNN) is a deep learning algorithm ideally suited to grid-based data, such as images. The most prominent and distinctive feature of a CNN is its ability to automatically and adaptively determine spatial hierarchies of low-level to high-level features, which it can use to discriminate between different classes of interest. Deep CNNs learn hierarchically; by learning more refined features at successively deeper layers. The initial layers extract general features, whilst the deeper layers focus on extracting problem-specific features^23^. The key to effective transfer learning is its ability to utilise the generalised characteristics of the initial layers and thus focus only on optimising the deeper layers, thus requiring fewer data and less time to learn new information. This is an instrumental approach in domains with few data and training resources available.

### Source and target defects

**Fig. 3:**
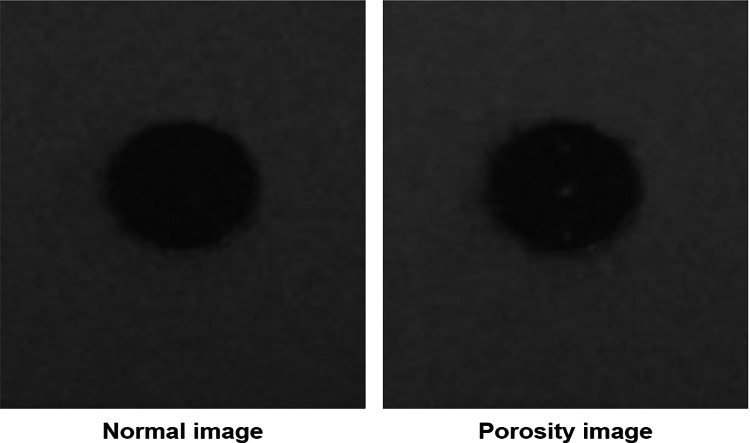
A normal vs porosity image example.

The experiments aimed to identify surface deformation defects by using the model trained on a porosity data set. An example of a normal and a porosity powder bed image from the source data set is shown in Fig. 3. The normal image on the left shows a dense (no porosity) cross-section of cylinder B2 at layer 753. The image on the right shows the cross-section of cylinder B2 at layer 376, depicting porosity.

**Fig. 4:**
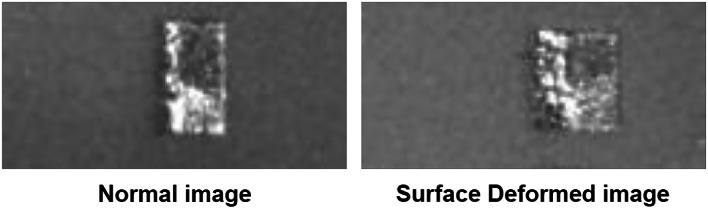
A normal vs surface deformed image example.

The target data set consisted of normal and deformed images. An example of a normal and a deformed image is shown in Fig. 4. The image on the left shows a normally printed cross-section at layer 439. The test specimen has a clear boundary and no deviation in the shape or form of the 3D object. The defective image on the right is the cross-section image at layer 445. Due to the unique geometry of the 3D object, the melt pool spread due to the temperature difference and resulted in a deformed and deviated finish. Surface deformation defects are a collection of defects stemming from a single source. Surface roughness, surface deformation, geometric deviations, warping, and a part growth near the edges are collectively related to surface and geometric inaccuracies in 3D printing.

### Data labelling

For accurate dataset labelling, we utilized both powder bed images (captured before and after laser exposure) and Optical Tomography (OT) images. Deformation was identified by two key indicators: the appearance of blue/cold spots on the OT images, and the partial visibility of the previously printed layer on the powder bed images after powder recoating. These signs reliably indicated deformation. The test specimens were intentionally designed with an overhang geometry to induce a temperature gradient, which led to an unstable melt pool and, consequently, deformation in the overhang region. The labelling process was thoroughly validated through a combined analysis of both powder bed and OT images. Further details on the labelling process can be found in our previous paper^9^.

## Methodology

The experiments were carried out on a high-performance computing machine with 64GB memory, Intel Core i5 @ 4.10GHz CPU and 24GB dedicated INVIDIA GeForce RTX 3090 graphical processing unit (GPU).

### Explanation of the primary model

The primary (or base) model used in this research was trained from scratch. We trained a deep convolutional neural network model on an LPBF image data set which contained porosity defects. The model went through intensive hyper-parameter tuning, various model architectures, data labelling and pre-processing data stages. The final model achieved an accuracy of 97% on the porosity data set with a 90+% precision and recall score. A summary of the model is presented here and is used as the base model for transfer learning on the new surface deformation data set^6^.

The base model (referred to as the porosity model from this point onwards) consisted of three convolutions and three max-pooling layers, followed by two dense layers. A variety of kernel numbers and kernel sizes were used in the model. The first convolution layer had 32 kernels of size $5 \times 5$. The second convolution layer had 80 kernels of size $3 \times 3$. Finally, the third and last convolutional layer had 48 kernels of size $2 \times 2$. The intuition for using different kernel sizes was to capture the various features from the input images. The first and third convolutional layers used the ’tanh’ activation function, whereas the second used the ’elu’ (Exponential Linear Unit) activation function. The first and second max-pooling layers had stride windows of size $4 \times 4$, and the last pooling layer had a stride window of size $2 \times 2$. The dense layers used the ’relu’ (Rectified Linear Unit) activation function. However, the dense layers were not used in the transfer learning approach as these were replaced by new dense layers for learning the target data set. The porosity model was trained on a balanced data set consisting of 2578 non-porosity and 2557 porosity images.

### Transfer learning settings on the primary model

**Fig. 5:**
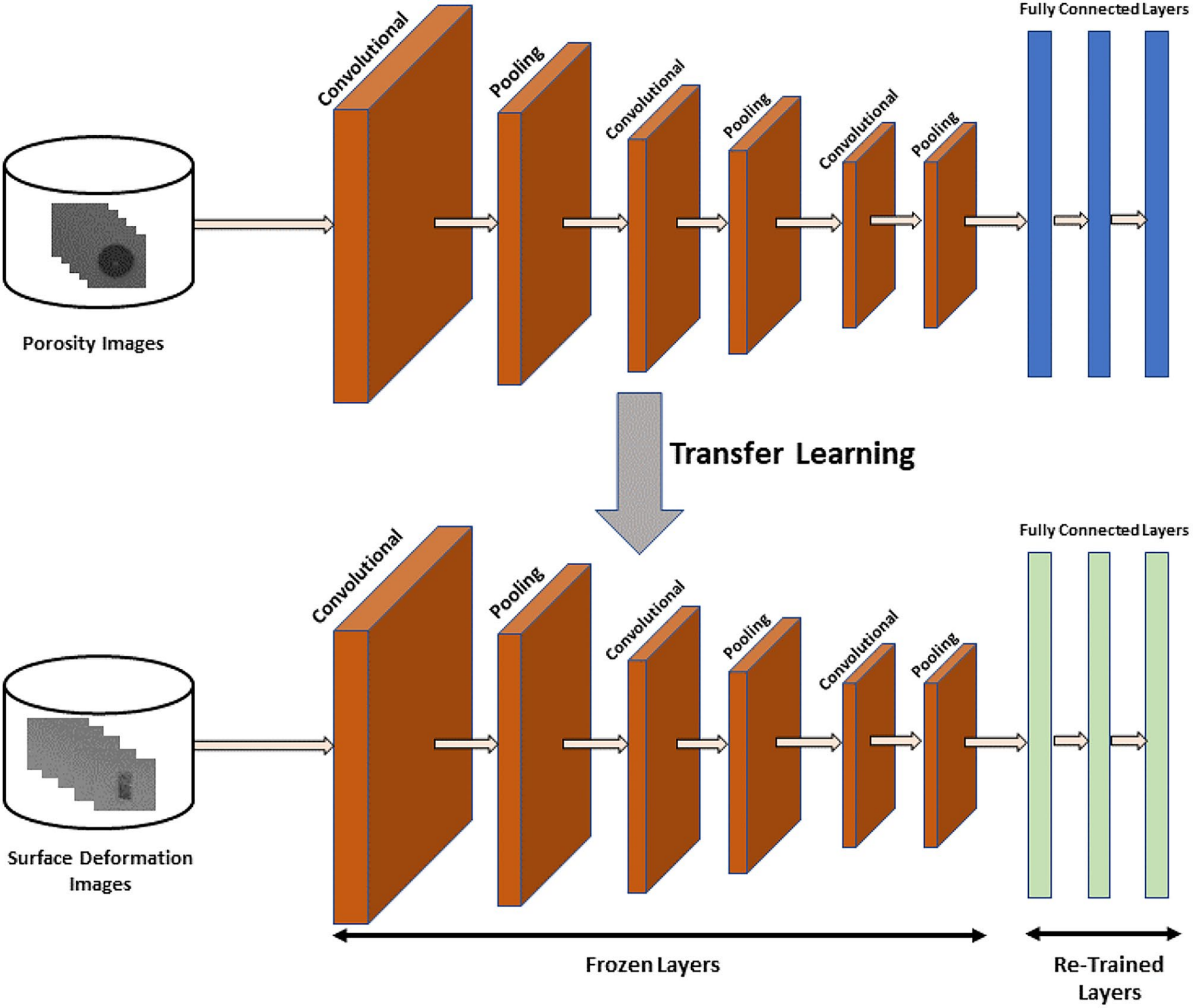
Transfer learning setting on the custom porosity pre-trained model.

The task of the transfer learning approach is to identify surface deformation defects from a target data set. Figure 5 shows the transfer learning setting.

As the target surface deformation data set is small and we expect the data’s features to be similar to those determined by the porosity model, we freeze the convolution layers and replace (and train) only the dense layers. This requires significantly fewer parameters to train than would be the case for a CNN trained fully from scratch.

The pre-trained convolution layers extract high-level features from the surface deformation images using parameters optimised for the porosity data. These features then feed into new dense layers, which are optimised to map these features to their required classification targets. The modified porosity model used a softmax classifier and categorical cross-entropy.

### Data set and pre-processing

The source and target data sets were split into training, validation and testing data sets to allow for practical training and evaluation of the model. The validation set monitored the performance of the model’s learning on the training data and assisted in hyperparameter selection. At the same time, the test set was kept separate and used only once for the model’s evaluation after the training and fine-tuning had taken place.

The porosity source data were $190 \times 150$ in size. For the source model’s training, the data set was split into train, validation and test samples using stratified sampling, ensuring that a consistent class distribution among the data sets was retained. The exact number of images in the train, validation, and test samples of the porosity data set is shown in Table 1.

**Table 1:** Train-test split of the porosity data set (source data set).

	Train	Validation	Test	Total
Number of images	2516	1078	1541	5135
Percentage	49%	21%	30%	100%

### Image resizing

**Fig. 6:**
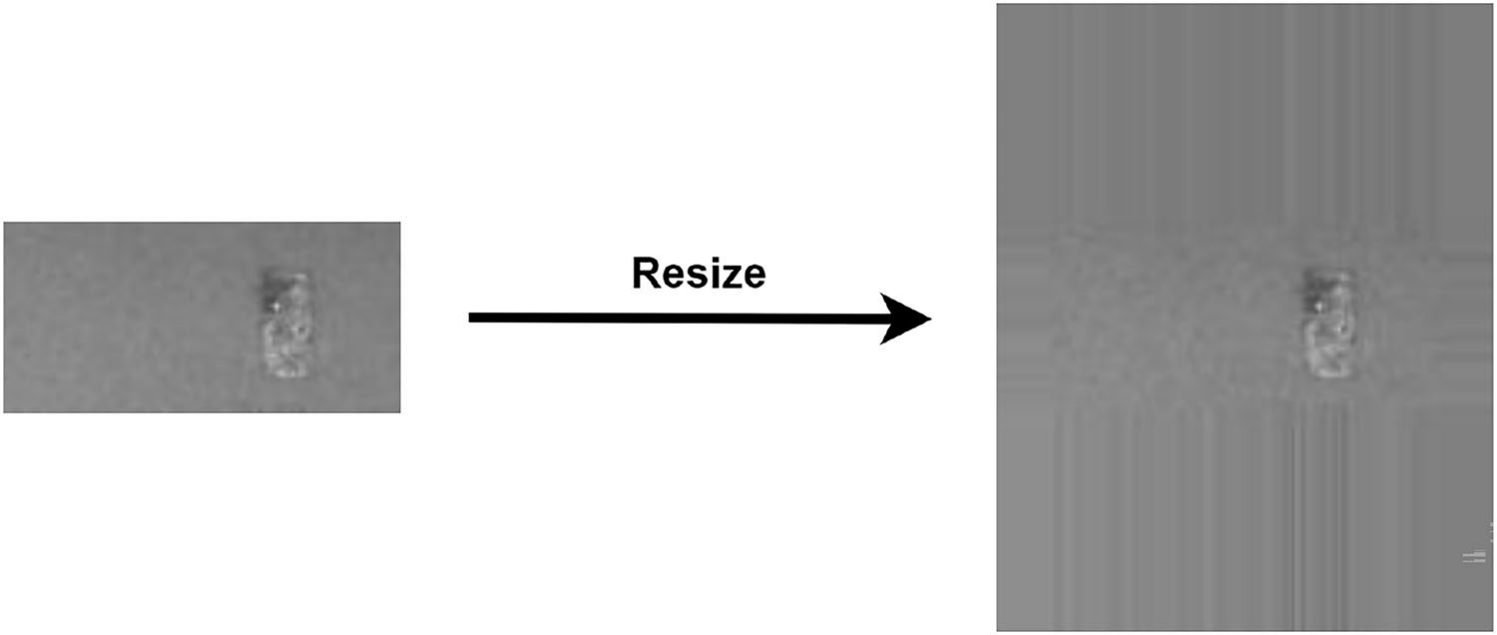
Resizing of the target images ($58 \times 120$), to that of the source image size ($190 \times 150$).

The surface deformation data set, (the target data set), had only 336 images in total. Of these, there were 168 defective and 168 non-defective images. The size of the target images ($58 \times 120$) was much smaller than the source images ($190 \times 150$). The surface deformation images were resized to the source image size of $190 \times 150$ by adding padding. The pixels at the borders (top, bottom, left, right) of the image were replicated to increase the image size. A sample, original image and the resized image are shown in Fig. 6. The test specimens laid at the centre of the powder bed images and were surrounded by unfused metal powder. Therefore, replicating the pictures’ border added more pixels that depicted the unfused metal powder. Moreover, both the porosity and surface deformation experiments were carried out with aluminium metal powder. This is why replicating the borders was chosen as the most suitable and natural choice for image resizing. The target images were split using the same approach as the source data set for fine-tuning the models.

### Fine-tuning settings

The primary model’s learning can be transferred via two methods, the fixed feature extraction method and the fine-tuning method. The fixed featured extraction method retrains only the new, fully-connected layers and keeps the feature extraction part frozen. In comparison, fine-tuning methods retrain all or a part of the feature extraction part of the model as well; depending upon the size and similarity of the target data set to the source data set^24^. As a rule-of-thumb, a data set is considered ’big’ if it has more than 1000 images per class. The surface deformation data has only 168 images per class. As far as data similarity is concerned, both data sets are very similar. Both data sets are powder bed images from two different builds following the same LPBF process, although the shape of the test specimens and the type of defects are different. However, most parts of the images consisted of unfused aluminium metal powder. In short, the two data sets are similar, so we can follow the fixed feature extraction method, where the target data set is small and similar to the source data set for fine-tuning via transfer learning.

The pre-trained, deep CNN porosity model’s feature extraction part was extracted and combined with new, fully connected layers. The entire feature extraction part was frozen and was not trained during the fine-tuning. The RMSprop optimiser was selected to train the dense layers with a small learning rate of 0.00002. The small learning rate ensured smooth convergence of the loss function. The mini-batch size was fixed to 10, as choosing a large batch could affect the model’s learning ability. Finally, the categorical cross-entropy loss function was selected.

### State-of-the-Art (SOTA) pre-trained models

Five SOTA pre-trained CNN models, MobileNet, VGG16, Xception, EfficientNetB1, and Inception ResNet V2, were selected for comparison. The feature extraction part of these models was frozen and used as-is for fine-tuning the dense layers. Further experiments were performed by retraining all the models’ 50% feature extraction part and comparing their performance based on the evaluation metrics. Additionally, data augmentation was used on the fly while training the models. Moreover, MobileNetV2, NASANet Mobile and ResNet50 were also trained on the surface deformation dataset. NASANet Mobile, MobileNetV2, and ResNet50 achieved an accuracy of 52%, 53% and 93% when trained by freezing their 50% feature extraction layers. However, their results were insignificant and therefore were not considered for further experimentation.

## Results and discussion

### Performance metrics and critical evaluation

Despite the small size of the target data set, the porosity model performed well and achieved an accuracy of 97%. Accuracy is an excellent evaluation metric when the test data set is balanced. However, it is prudent to test the model using various evaluation measures. The other evaluation criteria employed were confusion matrix, precision, recall and f1-score. The accuracy, precision, recall, f1-score and confusion matrix of the porosity model on the surface deformation data set are shown in Table 2. The unbiased model achieved excellent precision, recall and f1 scores for both classes. The recall for normal images was 96% and 98% for defective images, with only one miss-classification regarded as critical, being classified as belonging to the defective class. The confusion matrix showed that the fine-tuned model miss-classified only three surface deformation images.

**Table 2:** Porosity model’s results on target surface deformation dataset.

Model	Class	Accuracy	Precision	Recall	F1-Score	Confusion matrix	
Porosity model	Normal	97	0.98	0.96	0.97	49	2
	Defective	97	0.96	0.98	0.97	1	49

**Fig. 7:**
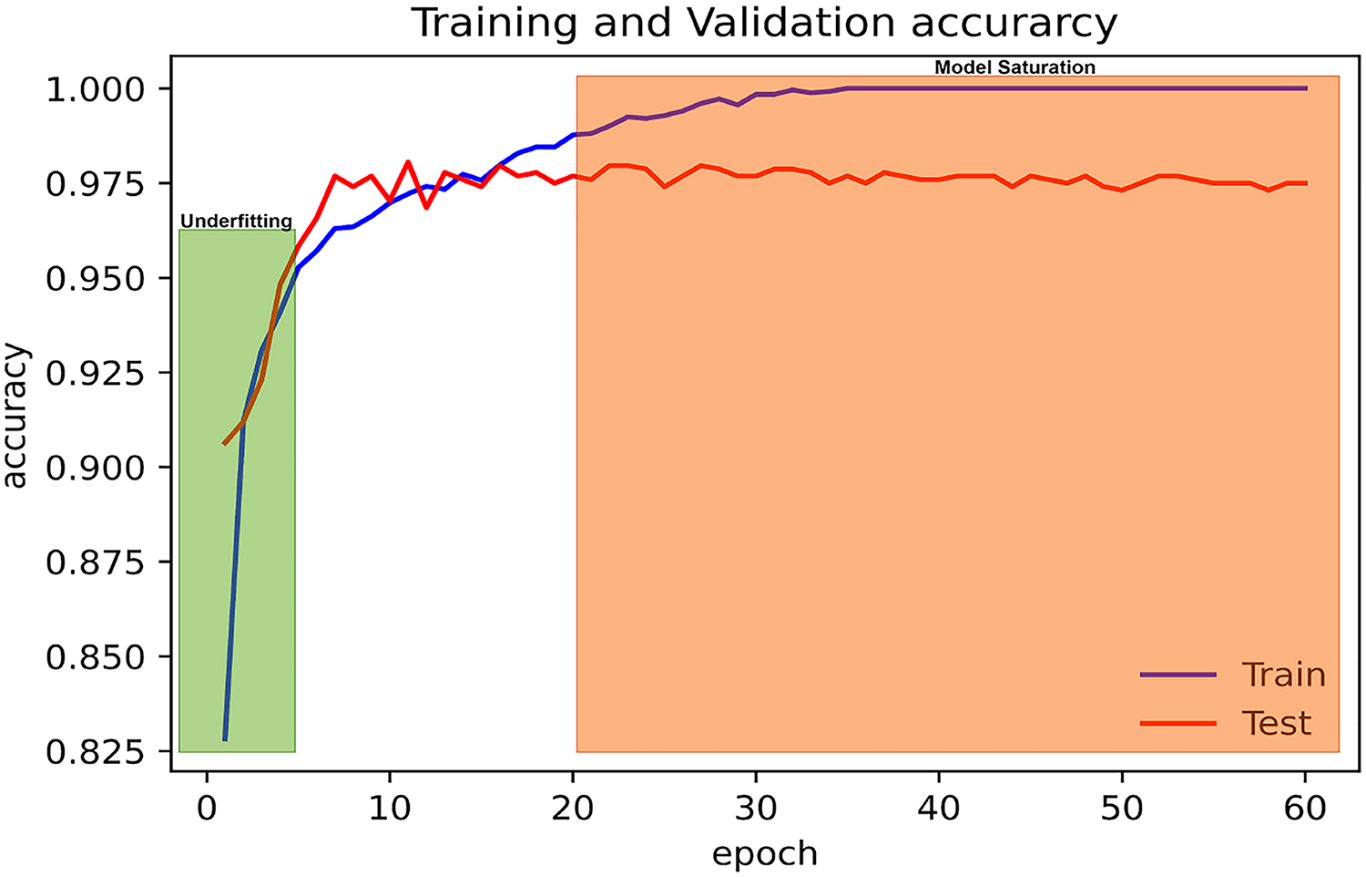
Under-fitting and over-fitting of the porosity model.

By plotting the model’s accuracy curves, the study ensured good training and identified any over-fitting or under-fitting of the model. Initially, the model was trained for 200 epochs with the early stopping of patience five on validation loss. However, it was observed that the model saturation reached its plateau after around 18–20 epochs, as shown in Fig. 7. This means the model is not learning anymore.

### Comparison with SOTA models

We compared the performance of the porosity model with the SOTA pre-trained CNN models. SOTA models were initially trained on the ImageNet data set and had millions of trainable parameters. These models were fine-tuned on the same settings as the porosity model to ensure unbiased comparison. The standard settings among the models were train and test split ratios, model evaluation metrics, loss function, optimiser algorithm, and batch size. Initially, the model’s feature extraction part was frozen, and only the classification layers were fine-tuned. Then, for a more detailed analysis, the models were also trained by unfreezing 50% of their feature extraction layers. The total parameters and total number of trainable parameters of each model at no training and 50% retraining of the feature extraction layers are shown in Table 3. The total trainable parameters were one of the criteria for selecting SOTA models. The total parameters of the SOTA models vary from a few million to up to 59 million. The inceptionResNetV2 model was the largest among them and had 59.05 million parameters. The smallest amongst the SOTA models was MobileNet, with 8.4 million parameters. The porosity model has only 476,578 total parameters.

**Table 3:** Total and Trainable parameters of Porosity model vs the SOTA pre-trained models for no training and 50% retraining of feature extractor.

Model		No Training	50% Training
	Total Parameters	Trainable Parameters	Trainable Parameters
Xception	36,590,890	15,729,410	30,587,674
VGG16	17,336,898	2,622,210	16,191,490
MobileNet	8,472,514	5,243,650	8,181,506
InceptionResNetV2	59,056,098	4,719,362	46,640,354
EfficientNetB1	16,406,409	9,831,170	15,948,442
Porosity model	**476,578**	**431,042**	**452,626**

Apart from the EfficientNetB1 model, all of the models performed well and achieved excellent results. In terms of accuracy, Xception, VGG16, MobileNet, InceptionResNetV2, EfficientNetB1 and the porosity model achieved an accuracy of 94%, 86%, 94%, 93%, 50%, and 97% respectively. The porosity model required only a fraction of the time, per training step, compared to the SOTA models. The custom porosity model only required 12ms per step for training and only 5ms for testing. The model outperformed the SOTA models and achieved the highest accuracy of 97% and recall of 98% for the critical class, the surface deformation images. Amongst the SOTA models, MobileNet and Xception acquired the highest results. However, unlike the porosity model, MobileNet and Xception were much denser, with 8.4 and 36.5 million parameters, 17ms and 40ms training time, and 14ms and 38ms testing time respectively, requiring significantly more time for training and testing. The porosity model had only 476,578 parameters and required a fraction of a second for training (12ms) and testing (5ms).

### Model visualisation

Deep learning models have reached unprecedented milestones in computer vision problems such as image classification, segmentation, image captioning and object detection. However, as most deep learning models work as a “black box”, there is significantly less information on how the model works. The input images are fed from one end of the model, and the classified images are gathered from the other end. Despite a good understanding of different layers, such as convolution, pooling and fully connected layers and their working, the actual decisive factors of the models are unknown. Due to this unknown factor of CNNs, it is almost impossible to debug why the model fails when it fails. This reduces the trust and reliability of deep learning models. In real-life applications, knowing how the model makes its decision and where it looks within the image for its decision-making process will help tremendously in building trust in artificial intelligence solutions. Moreover, it will help in the model’s debugging. Grad-CAM^25^ presents a visual explanation of a CNNs decision-making. Grad-CAM shows where the model looks for a particular class in an image. This facilitates seeing whether the model looks at the right image section and learns the relevant features. The model’s failed classifications or wrong predictions might be based on the wrong features. GRAD-CAM visualises the image area responsible for the model’s decision-making. Grad-CAM utilises the gradient of a class at the final convolution layer to produce a localisation map. The localisation map underlines the regions in the image where the model is looking to learn patterns for that specific class.

**Fig. 8:**
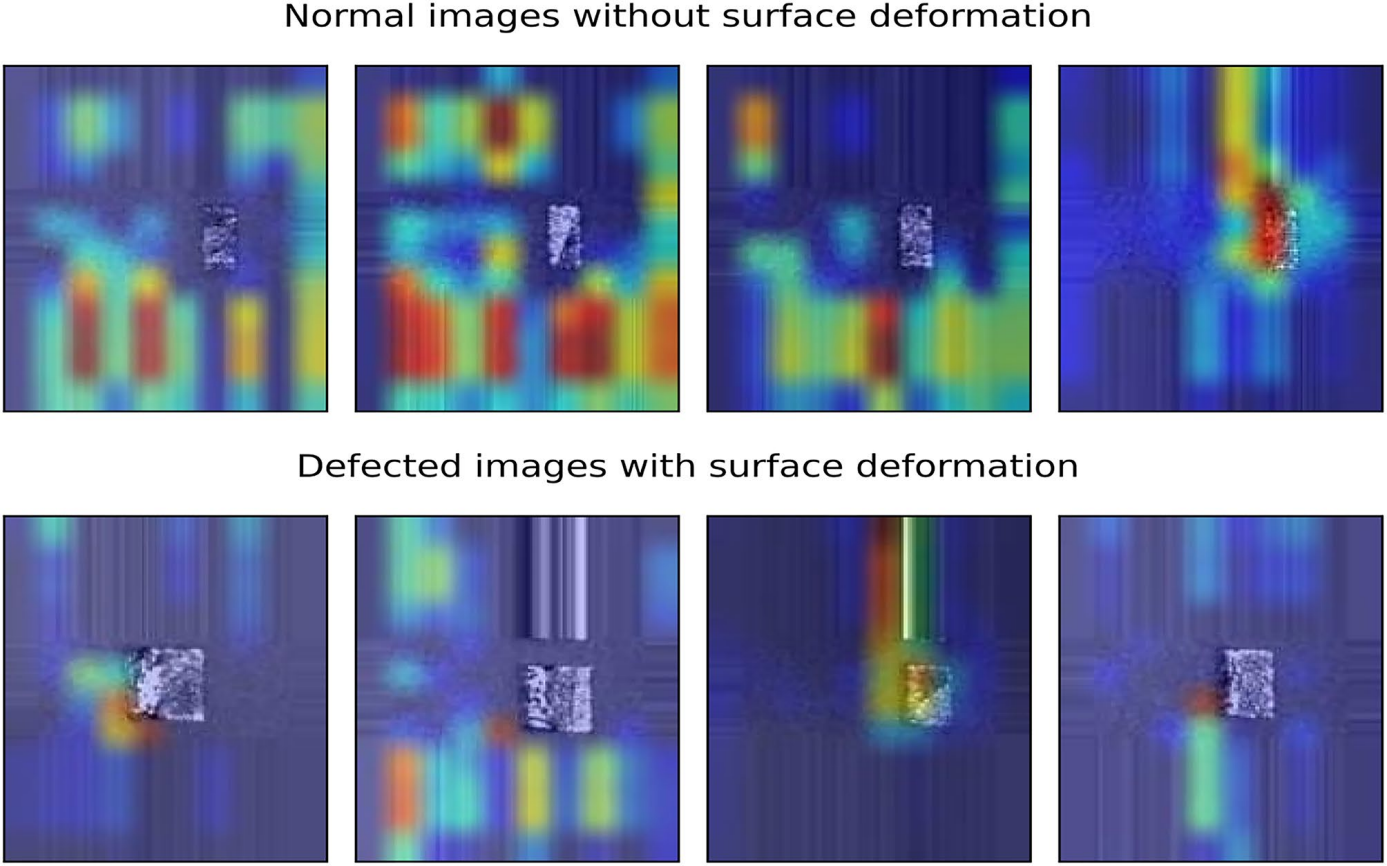
Grad-CAM visualisation of normal and defective images following the padded resizing method.

We employed Grad-CAM to visualise and understand how the model was working and to confirm that the model was learning the correct patterns. Grad-CAM highlights the portion of the image from where CNNs extract the decisive features for classification. The experiments aimed to detect surface deformation anomalies on and around the 3D test specimen. So, ideally, the model should make a decision about the surface being either deformed (defective) or normal based on the image features on and around the 3D object. The Grad-CAM visualisation of some normal and defective images is shown in Fig. 8.

**Fig. 9:**
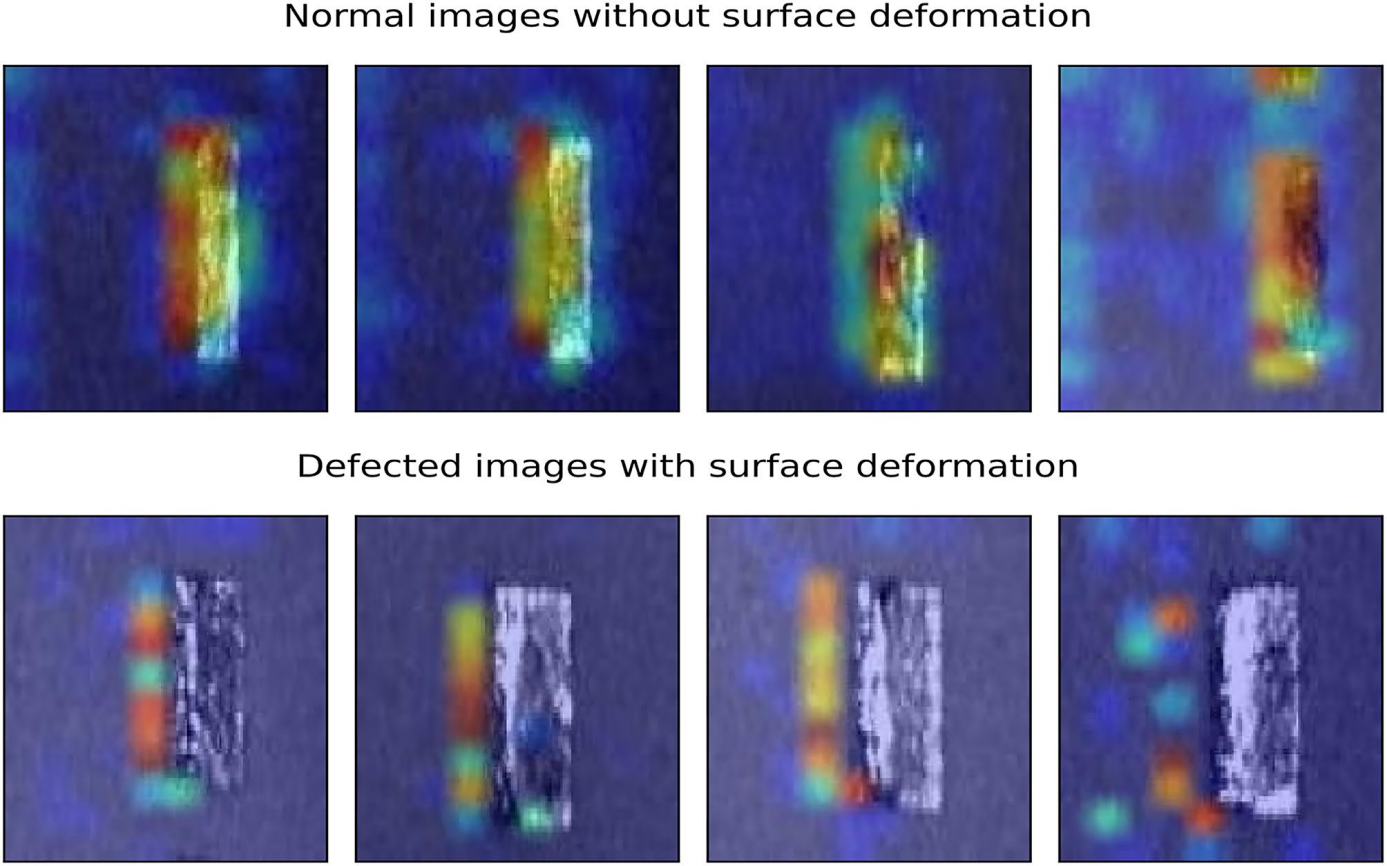
Grad-CAM visualisation of normal and defective images following the resizing method.

The Grad-CAM visualisation showed that the model was not learning the correct patterns and features from the input images. The model was clearly making most of its decisions based on the features extracted from the unfused powder part of the image and not the 3D test specimen. The GRAD-CAM visualisation showed that the model was learning features from the border region of images. Clearly, the model’s decision making capability should only be influenced by the features extracted from the 3D object under study. Although the accuracy and other evaluation metrics are excellent, the model’s decisive criteria are not what is expected, as the model mostly learnt features from the unfused powder with only a few from the actual test specimen. The problem arises from the image resizing strategy. The experiments resized the surface deformation images by duplicating the borders, which introduced lines and streaks in the images. These new features, lines and streaks are unique to each image, as each image might have different border pixels. Instead of learning the surface deformation from the 3D test specimen, the model learned these features and made decisions based on this newly induced noise in the images. This is unacceptable even though the model is performing excellently.

To rectify this problem, the image resizing strategy was changed from a padded border to a resizing approach using area interpolation. This approach generates resized images free of border extension noise and introduces no lines or streaks in the images. The experiments were repeated with the new, improved quality data set. Figure 9 shows the Grad-CAM visualisation of some normal and defective images of the new, resized surface deformation data set. The model is now capable of distinguishing surface deformation features from the test specimen in the images. The model’s learning is focused on the 3D test specimen instead of other patterns and features from the images. Although the model is not entirely looking at the test specimen, compared to the padded images, the model’s learning is much more reliable and realistic.

**Table 4:** Evaluation of the Porosity model versus the SOTA pre-trained models on the resized data set by freezing the entire feature extractor part.

Model	Training time (ms)	Testing time (ms)	Class	Accuracy	Precision	Recall	F1-Score
MobileNet	17	11	0	0.93	0.98	0.88	0.93
			1	0.93	0.89	0.98	0.93
VGG16	1046	39	0	0.91	0.94	0.88	0.91
			1	0.91	0.89	0.94	0.91
Xception	39	39	0	0.92	0.92	0.92	0.92
			1	0.92	0.92	0.92	0.92
EfficientNetB1	46	36	0	0.50	0	0	0
			1	0.50	0.5	1	0.66
InceptionResNetV2	1076	97	0	0.90	0.92	0.88	0.90
			1	0.90	0.88	0.92	0.90
Porosity model	**15**	**5**	0	**0.94**	**0.96**	**0.92**	**0.94**
			1	**0.94**	**0.92**	**0.96**	**0.94**

The training and testing times are per batch size of 16. The experiments were carried out on a high-performance computing machine with 64 GB memory, Intel Core i5 @ 4.10GHz CPU and 24GB dedicated INVIDIA GeForce RTX 3090 GPU.

**Fig. 10:**
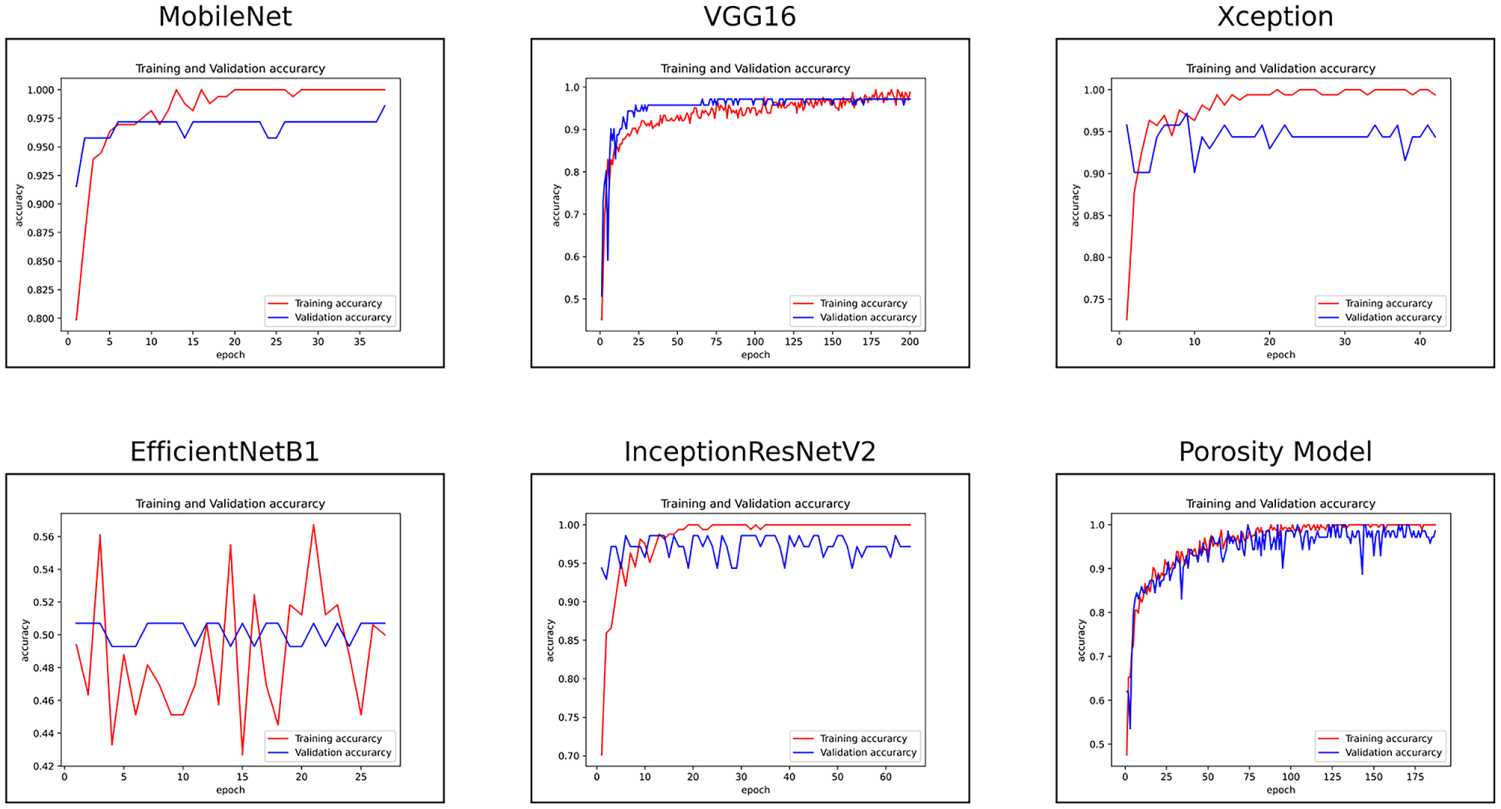
Training learning curves of all the models on training and validation data sets with resized images by keeping their feature extraction part frozen.

A comparison of the porosity model and the SOTA models on the new, improved data set is shown in Table 4. The porosity model is still the best-performing model with the highest accuracy of 94%. The feature extraction part of the porosity as well as the SOTA models were frozen, and only the dense layers were retrained. Apart from EfficientNetB1, all the SOTA models achieved 90% plus accuracy. MobileNet, VGG16, Xception, and InceptionResNetV2 models achieved an accuracy of 93%, 91%, 92%, and 90%, respectively, with excellent precision and recall scores. The porosity model is still the most time-efficient, lightweight, compact, and most suited for deployment in practical scenarios in terms of training and testing time. The training accuracy learning curves of all the models are shown in Fig. 10. Comparing the training learning curves of the porosity model with the best-performing SOTA models, all of the models showed generalised and unbiased learning.

Experiments were also conducted by unfreezing 50% of the feature extraction part of all the SOTA and the porosity model. A comparison of the porosity model and the SOTA models on resized surface deformation using 50% retraining of the feature extraction part is shown in Table 5. The performance of the porosity model decreased when trained using the 50% retraining of its feature extraction part. This means the knowledge transferred from the porosity data set was more useful in identifying the surface deformation defect. However, the porosity model still achieved an excellent accuracy of 93% and a recall of 94%. Xception acquired the best accuracy of 96% while VGG16 and MobileNet both achieved 95% accuracy. VGG16 acquired the highest recall of 100% among all the models. The performance of the EfficientNetB1 increased when trained by unfreezing 50% of its feature extraction layers. The accuracy of EfficientNetB1 increased from 50% to 82% when trained using 50% of its feature extraction layers.

**Fig. 11:**
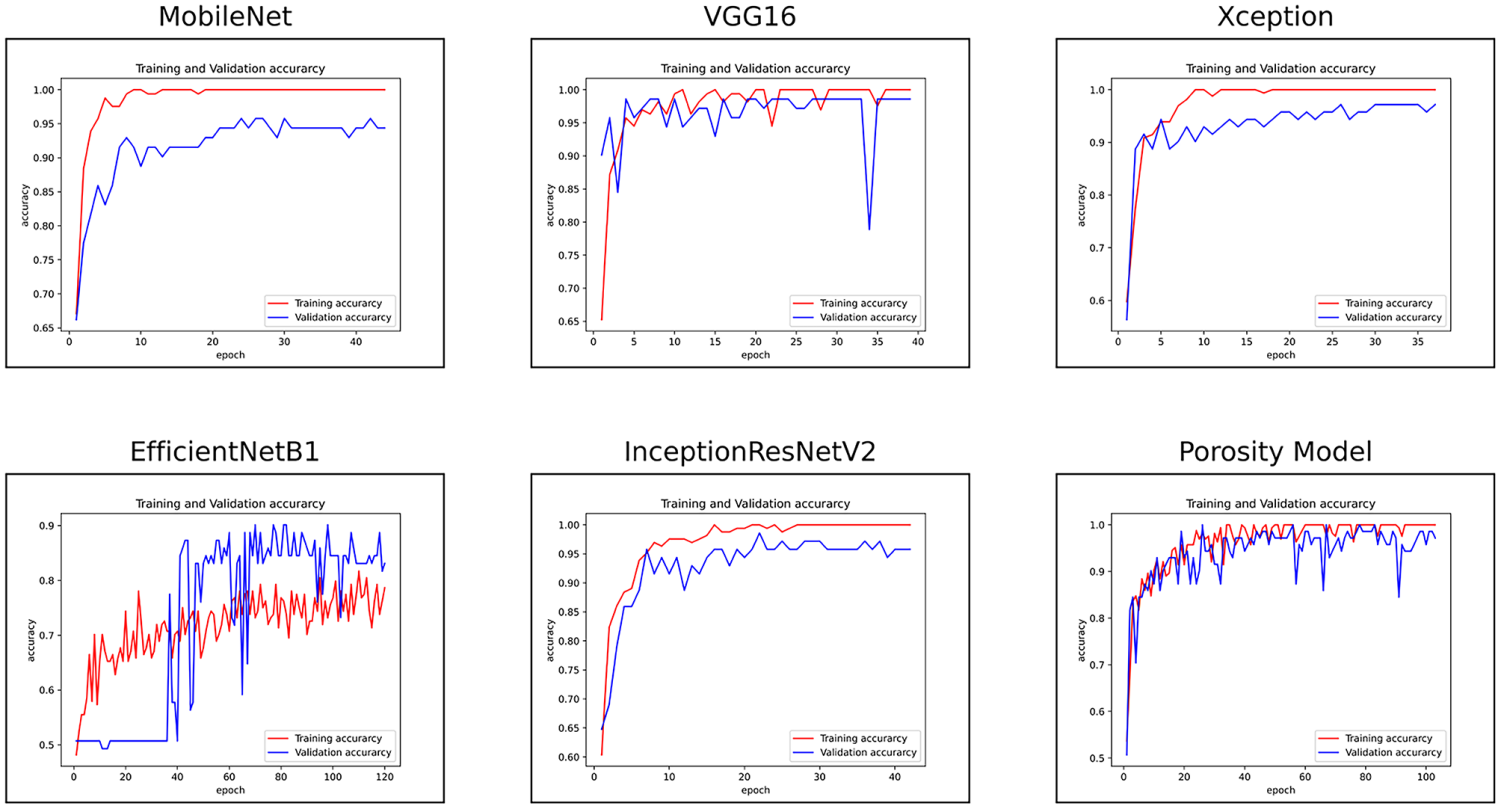
Training learning curves of all the models on training and validation data sets with resized images using 50% retraining of the feature extraction part.

The experiments showed that the SOTA models could identify surface deformation defects by retraining their deeper feature extraction layers. This means that the SOTA models have to unlearn some of the knowledge they learned from the ImageNet data set and retrain new surface-deformation data sets in order to distinguish between normal and surface-deformed images. On the other hand, the proposed porosity model can successfully transfer all of its knowledge learnt from similar LPBF problems with minimum retraining required and achieve the best result of 94% accuracy when trained only on the dense layers. There are several key similarities between the seeded porosity dataset and the surface deformation dataset. From an image processing perspective, both datasets share the same pixel range, as they involve Aluminium powder, and a significant portion of each image is covered by unfused metal powder. Moreover, the printed parts in both datasets display similar colour characteristics for the fused/printed regions. The main distinction lies in how porosity and surface deformation defects appear in the images. Because of these common visual elements, a model trained on the porosity dataset is already familiar with critical features, such as the presence of unfused metal powder and the appearance of printed regions. This familiarity enhances the model’s ability to transfer knowledge when learning to detect surface deformation defects. However, it’s important to note that noise or recurring patterns in the unfused powder could potentially distract the model from focusing on the relevant features, as demonstrated in Fig. 8 of the ’Model Visualisation’ subsection. In contrast, pre-trained models like those trained on large datasets such as ImageNet-which consist of a wide variety of objects-offer a broad foundation of visual patterns and general features. While this versatility is beneficial, these pre-trained models are unfamiliar with LPBF-specific defects. All the SOTA models achieved 90% plus accuracy except EfficientNetB1. The superior performance of the SOTA models over the porosity model is at the cost of more retraining of their feature extraction layers and at the cost of more training and testing time. The porosity model is still the most time-efficient in terms of training (16 ms) and testing time (5 ms), lightweight, compact, and most suited for deployment in practical scenarios in terms of training and testing time. The training accuracy learning curves of all the models are shown in Fig. 11, and all of the models showed generalised and unbiased learning.

**Table 5:** Evaluation of Porosity model versus the SOTA pre-trained models on the resized data set by freezing the top 50% feature extractor part.

Model	Training time (ms)	Testing time (ms)	Class	Accuracy	Precision	Recall	F1-Score
MobileNet	31	14	0	**0.95**	0.94	0.96	0.95
			1	**0.95**	0.96	0.94	0.95
VGG16	1084	39	0	**0.95**	1	0.90	0.95
			1	**0.95**	0.91	1	0.95
Xception	1077	39	0	**0.96**	0.96	0.96	0.96
			1	**0.96**	0.96	0.96	0.96
EfficientNetB1	1097	37	0	0.82	0.75	0.96	0.84
			1	0.82	0.94	0.68	0.79
InceptionResNetV2	2164	96	0	0.91	0.94	0.88	0.91
			1	0.91	0.89	0.94	0.91
Porosity Model	**16**	**5**	0	**0.93**	**0.94**	**0.92**	**0.93**
			1	**0.93**	**0.92**	**0.94**	**0.93**

The training and testing times are per batch size of 16. The experiments were carried out on a high-performance computing machine with 64GB memory, Intel Core i5 @ 4.10GHz CPU and 24GB dedicated INVIDIA GeForce RTX 3090 GPU.

### Comparison with similar studies

The results and various solution features of our experiments are compared with three relevant studies. A comparison is shown in Table 6. All the relevant studies used pre-trained models.^19,20^ has pointed out the need for shallower or lightweight models. Our approach bridges this gap and proposes a purpose-built, lightweight CNN-based solution instead of a complex, general-purpose, pre-trained model.^18^ achieved the best accuracy of 99.27%. However, they only considered one defect, porosity. Moreover, the excellent resolution of their camera system (3.69µm/pixel) resulted in high-definition images. On similar lines,^20^ used a high-definition camera system (6µm/pixel). The performance of image processing solutions relies significantly on the quality of the images. The experiments conducted in this paper achieved better results than^19^. However, the relatively poor camera resolution (200µm/pixel) severely hampered the images’ quality and resulted in slightly lower accuracy. That said, the proposed solution is more realistic and promising as high-resolution cameras are not prominent in the industry, and most in-built cameras in printers are usually of low resolution. The proposed solution was tested in a more realistic setting and achieved excellent results, confirming the correctness of the experimental approach. The novelty of the work is that no other study has attempted to transfer knowledge from one defect to another.^18^ had used a pre-trained model, VGG-16, to identify porosity. On the same lines,^20^ had also employed the pre-trained model Xception to identify various defects.^19^, on the other hand, had tried to transfer knowledge from one metal powder type (316L) to another (CuSn8). Their best accuracy was 85% with VGG-16 and 87% with ResNet-18 pre-trained models. This work has attempted a more challenging problem, the transfer of knowledge between two defects, and achieved a much better accuracy of 94% when trained only on dense layers keeping the feature extraction layers frozen. The results proved the correctness of the methodology and opened a new stream of research with far more practical benefits.

**Table 6:** Comparison with relevant research studies.

	Our model	Paper by^18^	Paper by^19^	Paper by^20^
Pre-trained model	–	VGG-16	VGG-16 ResNet-18	Xception
Data type	Powder bed images	Powder bed images	Acoustic emissions signals	Powder bed images
Image resolution	200 micro-meter/pixel	3.69 micro-meter/pixel	–	6 micro-meter/pixel
Data size	**10593**	8904	8000	1356
Target defects	Porosity and surface deformation	Porosity	Balling, lack of fusion, keyholes, conduction mode	Balling, incomplete spreading, spatter, groove, ridge, scatter powder

## Conclusion

This study has successfully shown that transfer learning can be effectively applied to quality assurance in 3D metal printing, particularly in detecting porosity and surface deformation defects using Laser Powder Bed Fusion (LPBF) datasets. We successfully demonstrated transfer learning on two challenging LPBF defects, porosity and surface deformation. To the best of our knowledge, no other study has attempted to employ transfer learning between two LPBF defects. The porosity model outperformed the SOTA and achieved an excellent accuracy of 94% when trained only on dense layers and acquired generalised and unbiased learning. However, the accuracy of the porosity model dropped to 93% when trained by unfreezing 50% of its feature extraction layers. The custom-developed model achieved excellent results while requiring fewer parameters and significantly reducing both training and testing times compared to SOTA models. This achievement underscores the practical advantages of deploying a more compact, efficient model in real-time LPBF processes, where speed and resource are critical. Our research further validates the potential of transfer learning in the field, demonstrating that knowledge gained from one defect type can be successfully adapted to identify another, despite limited data. This approach not only confirms the versatility of CNNs across different domains but also highlights the importance of developing resource-efficient AI models for industrial applications, especially in IoT contexts where hardware capabilities are constrained. By achieving remarkable accuracy with a significantly reduced dataset and demonstrating the model’s ability to learn critical features for defect identification, this study paves the way for future advancements in in-situ monitoring and control of 3D printing processes, emphasising the strategic importance of transfer learning in overcoming data scarcity and complexity in capturing LPBF data.

## Data Availability

The datasets generated during and/or analysed during the current study are available in the Surface_Deformation_Data repository, https://github.com/ayub-ensari/Surface_Deformation_Data.
